# Application of the electrical resistivity tomography in groundwater detection on loess plateau

**DOI:** 10.1038/s41598-023-31952-7

**Published:** 2023-03-24

**Authors:** Jiaqi Wu, Fuchu Dai, Pan Liu, Zhiquan Huang, Lingchao Meng

**Affiliations:** 1 College of Geosciences and Engineering, North China University of Water Resources and Electric Power, Zhengzhou, 450046 China; 2 Key Laboratory of Urban Security and Disaster Engineering of Ministry of Education, Beijing University of Technology, Beijing, 100124 China; 3 School of Computer and Information Engineering, Luoyang Institute of Science and Technology, Luoyang, 471023 China

**Keywords:** Hydrology, Natural hazards

## Abstract

Agricultural irrigation of the South Jingyang tableland in Shaanxi Province, China has led to a continuous rise of the groundwater level and has triggered a series of loess landslides, thereby seriously affecting the life and property safety of local residents. Research shows that the major cause of the landslide in the loess layer of the South Jingyang tableland is the rising groundwater level. Therefore, the research on the formation mechanism of landslide in this area should include the investigation of the stratigraphic structure and groundwater level distribution characteristics. On this basis, a series of approaches, such as electrical resistivity tomography (ERT), borehole, and laboratory tests, was carried out on the South Jingyang tableland, and the groundwater level distribution and stratigraphic structure in the study area were determined. The qualitative relationship between resistivity value and water content at different depths was detected using the inversion results of ERT and borehole data. Through laboratory tests, the quantitative relationship between resistivity values under different water contents was established. The precise depth of the groundwater level was inferred by connecting the qualitative relationship with the quantitative relationship, and then a detailed 3D geological model was established by linking the inversion results of ERT with the field borehole lithology data and geological survey data. The detection results show that when the qualitative and quantitative analyses of the ERT inversion results were combined, the distribution of the groundwater level was accurately judged. The ERT is effective in reflecting the stratigraphic structure and hydrological characteristics of the Loess Plateau, and its potential as a supplementary technology for detecting the groundwater level is reasonable. This study addresses the limitation and inaccuracy in determining the stratum structure and groundwater level by solely relying on borehole information or ERT. The established 3D geological model not only provides a basis for the study of groundwater table fluctuation, but also a technical guidance for the stability evaluation of loess slope, landslide prediction, and early warning in the study area.

## Introduction

In China, the distribution area of loess is 62.46 km^2^, accounting for 6.6% of its total land area. The Loess Plateau in the middle reaches of the Yellow River is the main distribution area of loess in China^1–3^. According to statistics, approximately 1/3 of landslides occur in the loess area^4,5^. The Loess Plateau of northwestern China is located in the middle reaches of the Yellow River, the most concentrated and largest loess region in the world^6–8^. The Loess Plateau developed in semiarid and arid environments, and large amounts of water have been pumped from the river for large-scale agricultural irrigation given that human activities have expanded into these regions^9,10^. The South Jingyang tableland in Shaanxi Province, China is a loess plateau area. Agricultural irrigation activities also increase in the region, with the development of economy. However, long-term irrigation and rainfall increase the infiltration channels, such as cracks and sinkholes on the edge of the tableland on the loess slope, directly or indirectly increase the groundwater level, and greatly impact the stability of the tableland slope. Such landslide hazards are usually distributed around the edge of the Loess Plateau, where irrigation is most intensive. Moreover, the area has a large number of landslide groups^11–13^; it generally shows an evidently gradual retrogressive trend ^14–19^. It is a typical irrigation-induced loess landslide. The occurrence of these landslides has caused serious losses to the lives and property of local residents.

The factors associated with the characteristics and mechanism of loess landslide, including strata lithology, slope shape, irrigation, and rainfall are discussed^20^. These factors affect the fluctuation of groundwater level in the slope. The traditional borehole method, which is used to explore the stratum distribution and groundwater level depth inside the loess slope in the south Jingyang tableland, not only destroys the soil inside the slope, but also entails high cost. Therefore, the non-invasive electrical resistivity tomography is used to detect the loess slope in the study area.

At present, electrical resistivity tomography is a broad-range subject of detection technology, which includes archaeology, environmental hydrogeology, geology, geotechnical engineering, and geomorphology^21^. It has the characteristics of large amount of data acquisition, high efficiency, low cost, rich inversion information, and easy interpretation. Ettore Cardarelli et al.^22^ conducted multidimensional (1D/2D and 3D) electrical resistivity survey of Rieti plain in central Italy to identify the internal stratum structure and lithology of landslide. The results indicate that 1D inverted resistivity models show bedrock depths in accordance with the borehole data and a complex subsurface layering of the overburden deposits. In addition, the 2D and 3D inversion data verify that the resistivity model retrieved by the electrical resistivity tomography not only evaluates the depth of bedrock and complex underground stratification in the study area, but also provides additional information, such as the continuity of bedrock and the existence of underground aquifer. Dominika and Stan et al.^23^ used the non-invasive and relatively rapid method of high-density electrical resistivity tomography in the ridge and the steep eastern slope of the Orlik massif to obtain a spatial image of a geological rock mass. Meanwhile, they combined the detection results and the results of mineralogical composition analysis to determine the nature and thickness of the weathered layers present on the study area. Khan et al.^24^ used the electrical resistivity tomography to determine the location and depth of crack zone in the highway slopes constructed with highly plastic clay soils at Texas. Qi et al. ^25^ introduced an improved high-density resistivity method based on network parallel technique and used this method to measure the 2D resistivity of a construction site at Yantai (Shandong Province, Eastern China). The measurement results demonstrated that the improved high-density electrical resistivity method is useful for rapid evaluation of subsurface geological structures in an urbanized area and provides more precise and faster data acquisition than conventional techniques. Hsu et al.^26^ used the 2D electrical resistivity imaging method to detect the contact between sediment and bedrock in a modern fluvial system. The results highlight the utility of the high-density electrical resistivity method in studies of landscape evolution, sediment transport, and sediment budgets. In hydrogeology, Ling et al.^27^ adopted the electrical resistivity tomography to investigate the internal structure of the Kualiangzi landslide, which is located in Sichuan province, China. The characteristics of the groundwater circulation were evaluated from variations in electrical resistivity and groundwater level. Uhlemann et al.^28^ conducted resistivity tomography survey of clay basin in water-rich area of Southeast Asia and analyzed the thickness change in clay layer and the increasing area of surface water entering groundwater. Hussain et al.^29^ used the electrical resistivity tomography to extract information about cavities, sinkholes, pathways for water infiltration, and the degree of karstification of underlying carbonate rocks.

In summary, the ERT has become a mature and reliable geophysical method in various geological engineering studies, but the study on building a 3D geological model by combining inversion results of the ERT with borehole data is limited. Therefore, resistivity tomography combined with limited borehole data reflects the stratigraphic structure and groundwater level distribution of the entire region. A 3D geological model construction is an important research direction. The current study draws on the achievements of previous scholars, uses the ERT to measure the South Jingyang tableland, and interprets and analyzes the measured data after inversion. Then, the laboratory test is carried out to obtain the quantitative relationship between the loess with different water contents and resistivity. Based on the inversion data and quantitative relationship, borehole data are combined to verify the effectiveness of the ERT, and the stratum structure, different soil thicknesses, and precise buried depth of groundwater level on the South Jingyang tableland are identified. Finally, through the identified geological information, a detailed visual 3D geological model of the study area is established. This method makes up for the limitation in determining the stratum structure and groundwater level by solely relying on borehole information. The research results not only verify the effectiveness of the resistivity tomography technology in the detection of groundwater level in the loess plateau, but also prove its potential as a supplementary technology for loess landslide investigation. Moreover, the established 3D geological model provides a research basis for analyzing the temporal and spatial variation law of the groundwater level of the loess slope, as well as a technical guidance and reference for slope stability analysis, prediction, and early warning.

## Geological background of the study area

**Figure 1:**
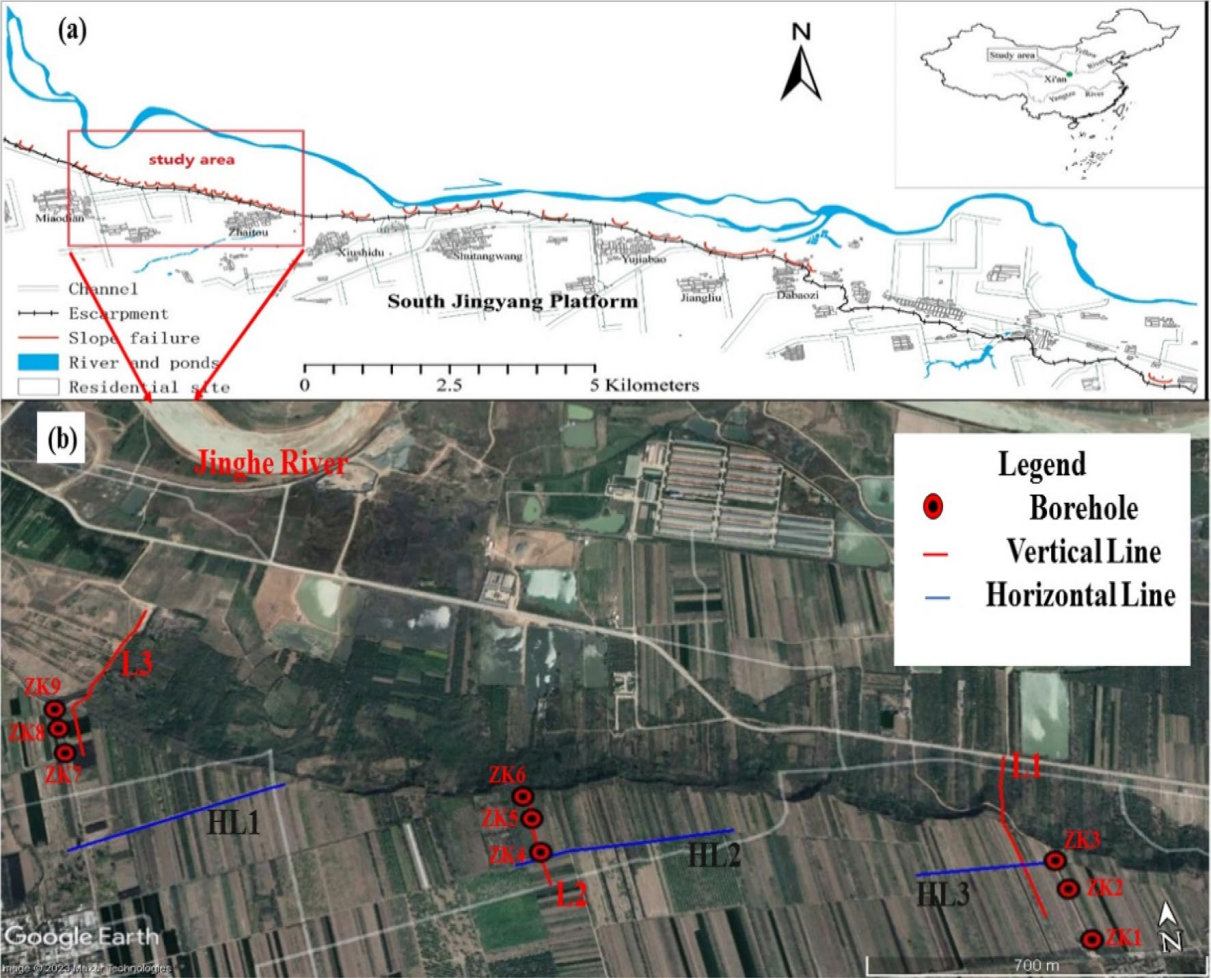
(**a**) Location map of the study area^13^ (**b**) location map of high-density survey lines and boreholes.

The study area is located on the South Bank of Jinghe River in Jingyang County, Xianyang City, Shaanxi Province, China, as shown in Fig. 1a; it belongs to the Weibei Loess Plateau in the Weihe Basin. Its geographical coordinates are 108° 46′ 04″–108° 53′ 12″ E and 34° 28′ 38″–34° 31′ 05″ N. The geomorphic types of the study area are mainly loess tableland and Jinghe terrace. The Loess Plateau is flat and broad, and the overall terrain is high in the northwest and low in the southeast. The elevation of the Loess Plateau is approximately 450–500 m. A scarp or steep slope with an overhead height of 40–90 m is formed at the edge of the Loess Plateau due to the gradual southward erosion of the Jinghe River. It has a gradient of approximately 45°–70°, and the shape is steep and has no primary soil support at the foot of the slope. The typical river terrace geomorphology provides a favorable condition for the initiation and movement of landslides in this region^13,30^.

**Figure 2:**
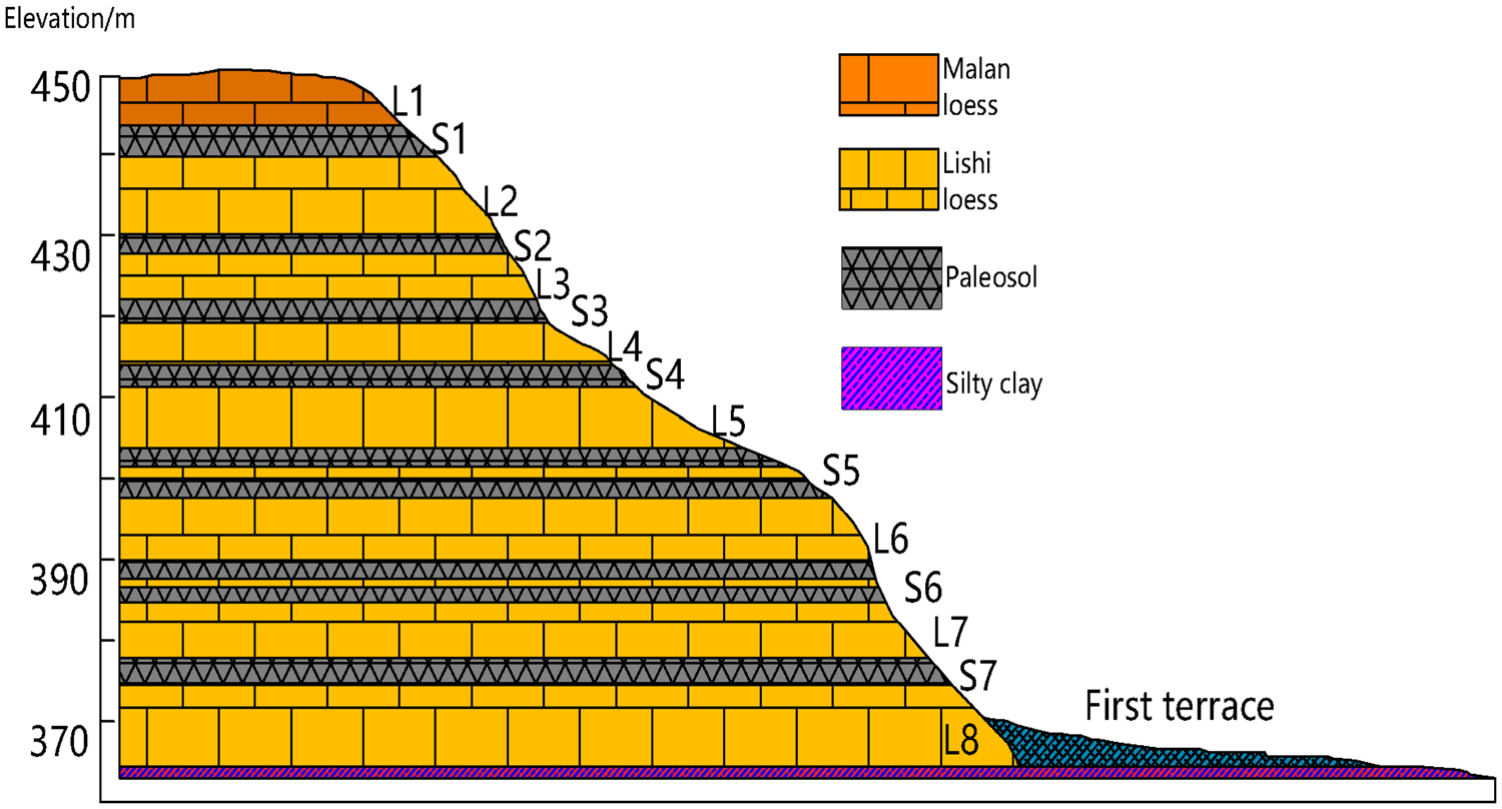
Vertical section of the stratum sequences.

In the study area, the outcropping conditions are better. The typical stratigraphic cross section with exposure quaternary strata in the study area is shown in Fig. 2. The upper part of the stratum is the Late Pleistocene Malan loess (Q3) with thickness of approximately 5–10 m, whereas the individual thickness of the underlying Middle Pleistocene Lishi loess (Q2) varies; its total thickness is approximately 50–70 m. Paleosol soils with individual thickness of approximately 2–5 m are sandwiched between the Middle Pleistocene Lishi loess and Malan loess. In the South Jingyang tableland, Lishi loess, and Paleosol soils constitute the main part of slopes.

Since the introduction of the water division project in the 1970s, more than 40 loess landslides have occurred in this tableland terrace slope topography. By the time of re-investigation in 1992, the groundwater level had increased by 13–37 m compared with that in 1976, and the average annual water level increased by 1.5 m. After irrigation, the water storage of the loess slope increased, resulting in a conspicuous increase in the groundwater level. This hydrogeological condition is an important factor that increases the occurrence of loess landslides in this region.

## Methodology

### Principle and method of ERT

Based on the difference in the electrical properties of underground medium, the electrical resistivity tomography, which delineates the stratigraphic structure and underground aquifer of underground space^25,26,31,32^, is carried out to detect the variation characteristics of spatial apparent resistivity in the stratum. In the survey process, the electrical resistivity tomography arranges survey electrodes and carries out geoelectric section survey. It has high survey efficiency, provides a large amount of data, and has a large amount of information, high observation accuracy, and fast speed. It is one of the most effective geophysical methods to find structural fracture and groundwater enrichment zones.

The ERT has various electrode arrangements, such as the Wenner array, the dipole array, and the differential array. The Wenner array has high vertical resolution and good horizontal stratification capability; it is more suitable for stratigraphic structure division and detection of stable aquifers^6,33,34^. It is the most common wiring method in field exploration. Therefore, the Wenner array is adopted for measurement in this detection. In the measurements using the Wenner array, the current supplied by electrodes A and B to the earth with the current intensity *I* is obtained, and then the measured current I and potential difference Δ V produced by the measurement electrode M and the measurement electrode N in the medium (underground rock and soil mass) are determined. The resistivity ρ between measuring electrodes M and N in the underground medium is calculated by formula (1), as follows:

1 $$ \rho = k\frac{\Delta v}{I} $$

In the equation: *K* is the coefficient of electrode device; Δ*V* is the potential difference; *I* is the current.

The theoretical conditions for application of Form (1) are as follows: the ground is an infinite horizontal surface, and the underground geological body is an isotropic medium.

However, the actual situation cannot completely satisfy the theoretical conditions. The terrain has certain ups and downs, and the underground medium cannot easily achieve isotropic homogeneity. In addition, in the actual detection process, the differences in equipment and the ground conditions of electrodes affect the collected data. Therefore, the resistivity values measured under these conditions do not represent the actual resistivity of rock and soil; they are referred to as apparent resistivity $\rho_{S}$, expressed as follows:

2 $$ \rho_{S} = k\frac{{{\Delta v}}}{I} $$

3 $$ k = \frac{2\pi }{{\frac{1}{AM} - \frac{1}{AN} - \frac{1}{BM} - \frac{1}{BN}}} $$

**Figure 3:**
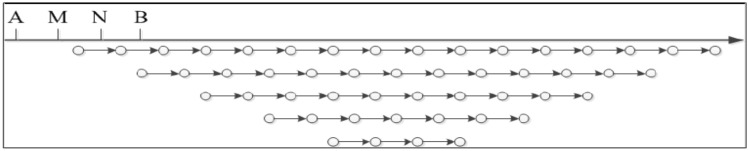
Sketch of data acquisition for Winner array.

Figure 3 shows a sketch of data acquisition for the Wenner array, that is, AM = MN = NB = *a*, where *a* is the electrode spacing. When measuring, points A, M, N, and B move toward the end point of the section at the same time, the apparent resistivity between M and N electrodes is measured, and the first layer apparent resistivity data are finally obtained. Subsequently, AM, MN, and NB increase at one electrode distance. A, M, N, and B move point by point to the end point of the section at the same time, obtaining the second layer apparent resistivity data. As the measurement proceeds, a section with an “inverted trapezoid” pattern is finally formed.

Through these principles, the edjd-3 resistivity tomography measurement system produced by Chongqing summit Geological Exploration Instrument Co., Ltd. of China was used for detection. The instrument has shown outstanding achievements in mineral exploration, energy exploration, geothermal exploration, urban geophysical exploration, environmental geological exploration, engineering geological exploration, as well as hydrogeological exploration, groundwater exploration, industrial and agricultural water use and domestic water use. The instrument abandons the traditional LCD display and keyboard control mode, realizes the operation of portable smart phone equipment, is convenient, supports dynamic display of multi-parameter measured curve, and integrates transmitting and receiving functions with small size and light weight. The instrument employs analog and digital multi-stage high precision filtering, signal enhancement technology and strong noise suppression technology to achieve extremely high small signal measurement accuracy, which enables the instrument to be better applied to work in high resistance areas and to obtain more stable and accurate measurement results.

According to the internal structure and hydrological conditions of the Loess Plateau of South Jingyang tableland in the references, six sections (Fig. 1b) are laid out to obtain the detailed characteristics of the electrical signals of the strata below the surface, three profiles (L1/L2/L3) of which are perpendicular to the sliding direction of the landslide due to the influence of site conditions, such as roads and scarps. Moreover, the other three (HL1/HL2/HL3) profiles are parallel to the edge of the tableland. The measuring line uses the Wenner array, and the interval between electrodes is 5.00 m. The coordinates of the electrode are located by RTK to determine the topographic changes on the tableland.

### Data processing of ERT

**Figure 4:**
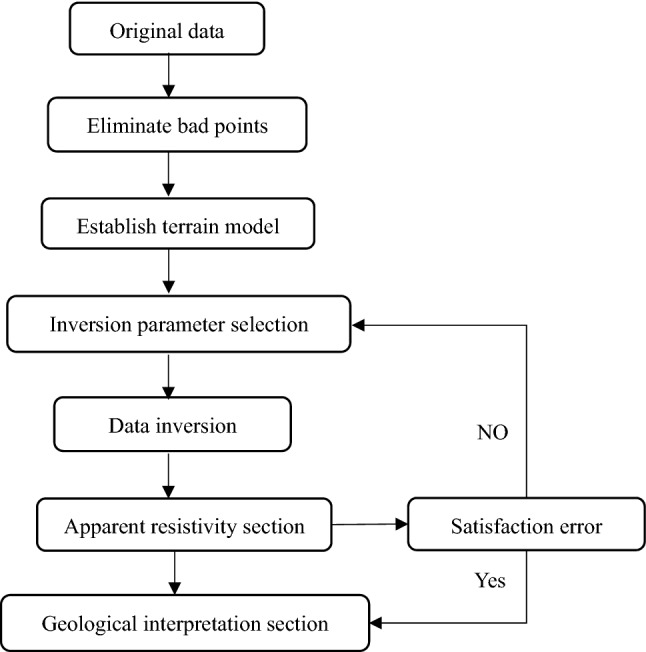
Flow chart of data processing.

At present, various electrical data processing software have been introduced at home and abroad, and Res2Dinv, Res3Dinv, and Earthimage series software developed by AGI Company are the most widely used. At present, Res2Dinv inversion software is the most widely used for the electrical resistivity tomography data processing in China. The software has mature theory, simple operation, and good mapping effect. Therefore, the 2D detection data in the current study are processed and analyzed by this software. For the original electrical data collected in the field, processing with Res series software mainly includes data reading and format conversion, bad point elimination, topographic correction, inversion parameter selection, inversion, and mapping. Figure 4 shows a flow chart of data processing.

### Principle of DDC-6 electronic automatic compensation instrument

DDC-6 electronic automatic compensation instrument is produced by Chongqing Geological Instrument Factory in China. It can be directly used for resistivity measurements. It adopts multilevel filtering, signal enhancement technology, and digital filtering. It has the advantages of strong anti-interference ability and high measurement accuracy.

**Figure 5:**
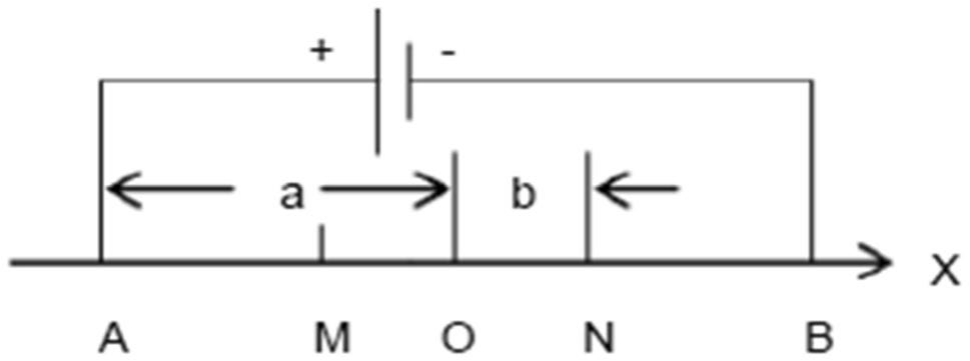
Schematic diagram of quadrupole vertical electrical sounding model.

In this study, the quadrupole vertical electrical sounding model in the instrument is carried out to measure the apparent resistivity of loess with different water contents in deep loess. The schematic of quadrupole vertical electrical sounding model is shown in Fig. 5.

The power supply current passes through the conductor and two electrodes A and B to establish a stable electric field of two opposite point power supplies. Based on the potential formula (the principle of potential overlap), any two points of M and N can be written directly, as follows:

4 $$ v_{m} = \frac{1 \rho }{{2\pi }}\left( {\frac{1}{AM} - \frac{1}{BM}} \right) $$

5 $$ v_{n} = \frac{1 \rho }{{2 \pi }}\left( {\frac{1}{AN} - \frac{1}{BN}} \right) $$

Potential difference between M and N:

6 $$ \Delta V_{MN} = V_{M} - V_{N} = \frac{1\rho }{{2\pi }}\left( {\frac{1}{AM} - \frac{1}{AN} - \frac{1}{BM} + \frac{1}{BN}} \right) $$

From the above formula, the following formulas for determining resistivity can be obtained:

7 $$ \rho = \frac{2\pi }{{\frac{1}{AM} - \frac{1}{AN} - \frac{1}{BM} + \frac{1}{BN}}}.\frac{{\Delta V_{MN} }}{I} = k\frac{{\Delta V_{MN} }}{I} $$

where

8 $$ k = \frac{2\pi }{{\frac{1}{AM} - \frac{1}{AN} - \frac{1}{BM} + \frac{1}{BN}}} $$

9 $$ a = OA = OB = \frac{AB}{2} $$

10 $$ b = OM = ON = \frac{MN}{2} $$

**Figure 6:**
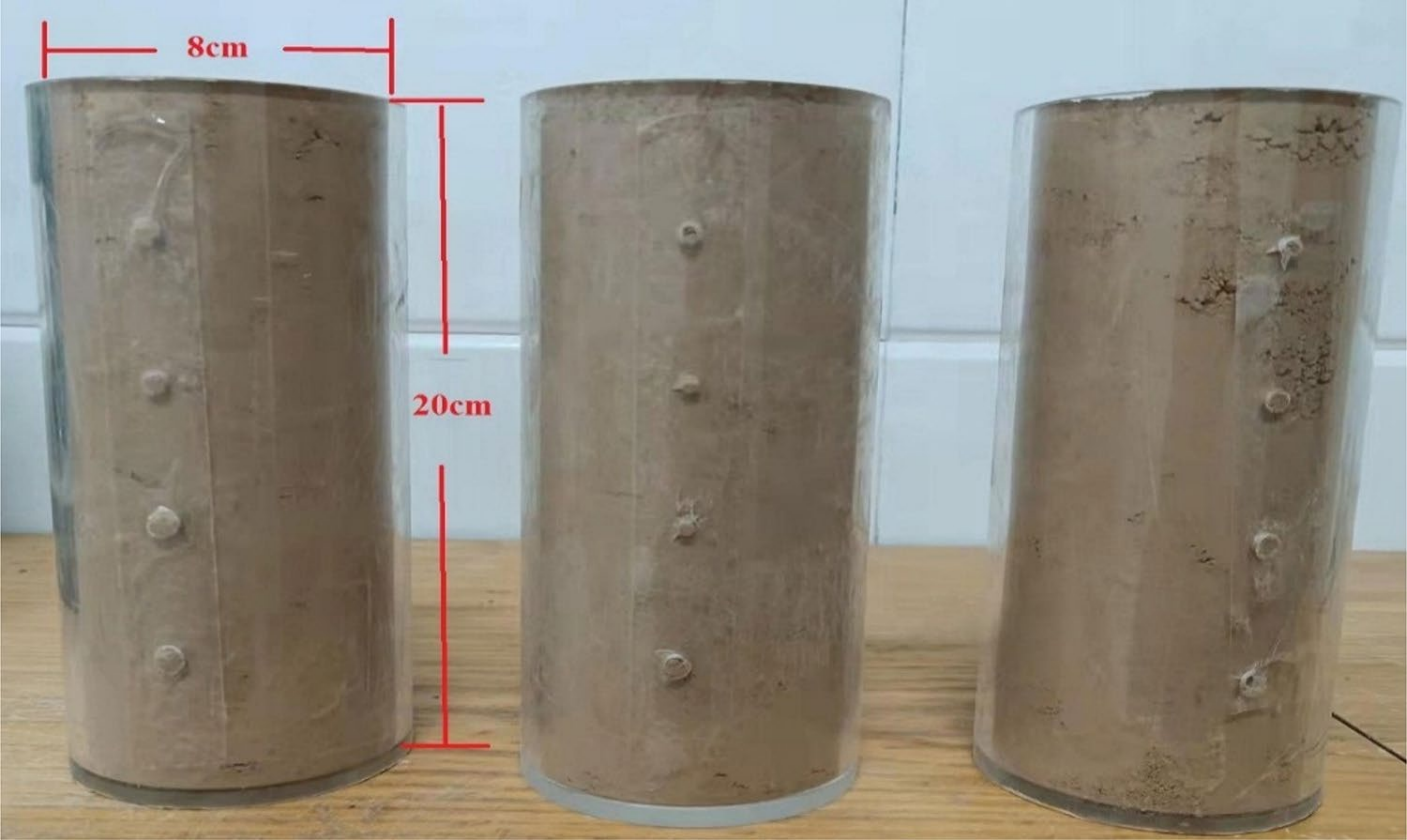
Reconstituted samples.

**Figure 7:**
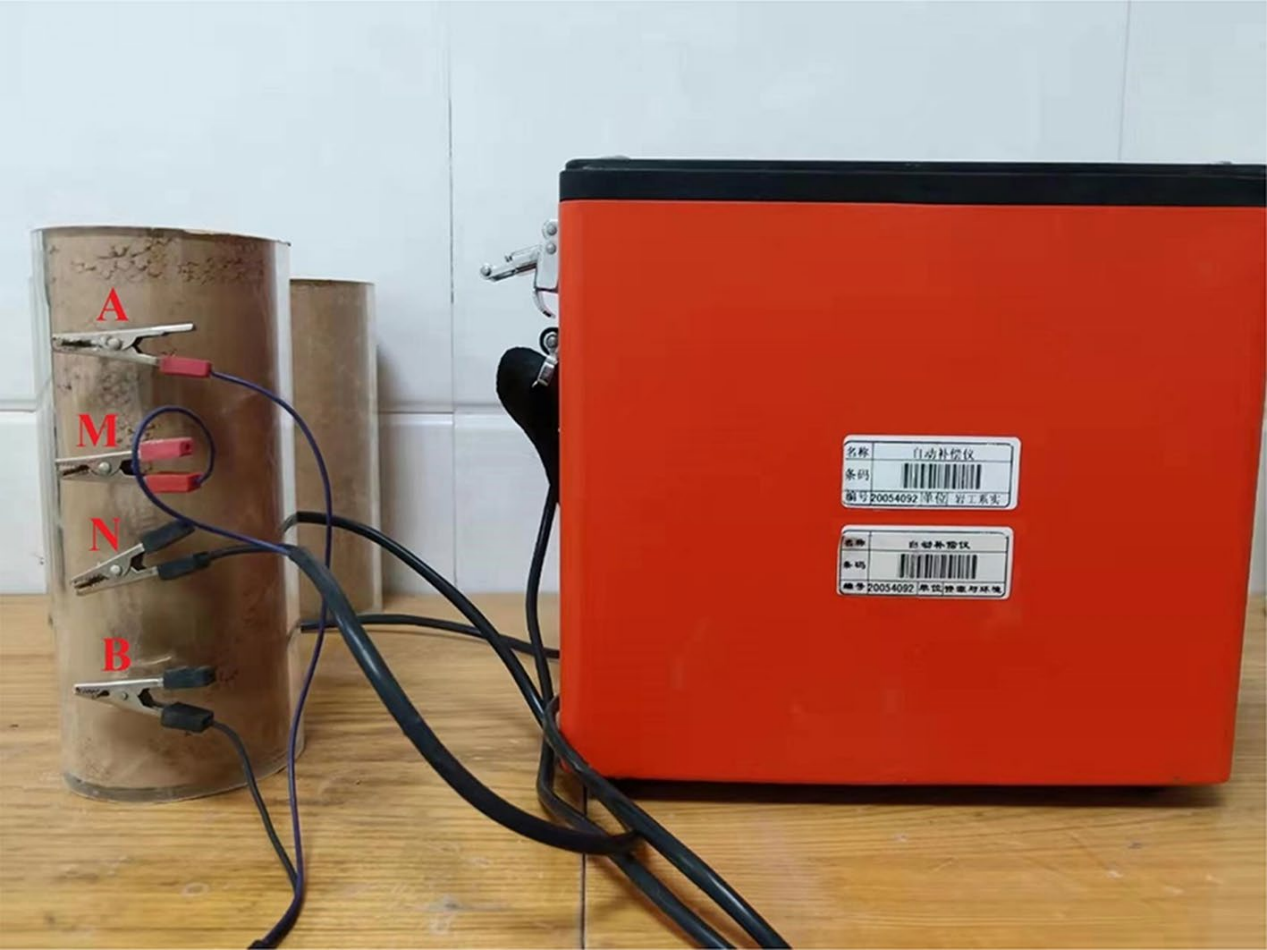
Test photo of DDC-6.

The original loess without root and other impurities are retrieved from the deep part of the site loess layer, and then dried and crushed sufficiently to prepare the reconstructed samples with different water contents. The samples with different water contents of 10–35% are equipped in special Plexiglas tubes (as shown in Fig. 6, Plexiglas tubes are nonconductors and do not affect the measurement results; they are easy to prepare) given that the liquid and plastic limit of loess in the study area is 13–30%. The height and diameter of the reconstituted sample in Fig. 7 are 20 and 8 cm, respectively.

According to the field measured data, the dry density of loess in this area is mainly between 1.43 and 1.53 g/cm^3^. All samples are equipped according to the dry density of 1.5 g/cm^3^ so that the variable of this experiment is only water content. The high mineralization of the aquifer in the study area greatly influences the resistivity of loess. The water with samples in the experiment was obtained from the shafts excavated during field irrigation to avoid errors. After sample preparation with irrigation water, the four electrodes A, B, M, and N are successively inserted into the sample and connected to the instrument according to the sequence in Fig. 5, as shown in Fig. 7. Then, the resistivity value of each sample is calculated according to the quadrupole vertical electrical sounding model. The resistivity of the three groups of samples corresponding to each water content is averaged as the resistivity value under this water content condition.

### Results and analysis

**Figure 8:**
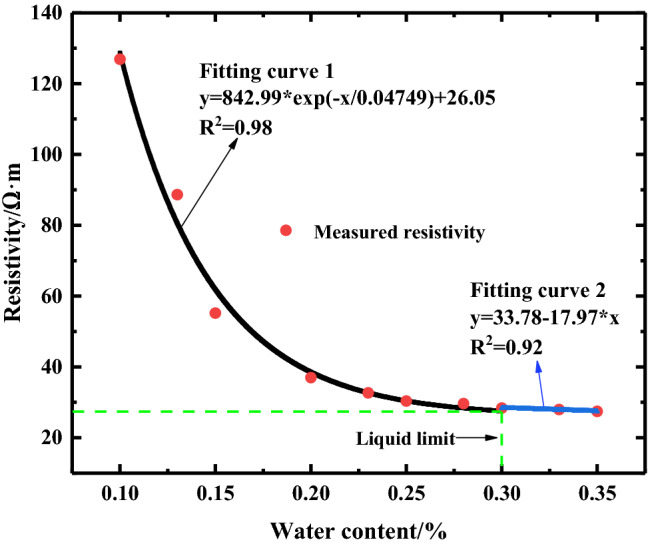
The characteristic curve of the relationship between water content and resistivity of loess.

The quantitative relationship between water content and resistivity of loess, which provides basis for groundwater level division, is determined through laboratory tests to obtain more accurate groundwater level data through resistivity profile. The characteristic curve of the relationship between water content and resistivity of loess in the study area is shown in Fig. 8. The figure shows that the loess resistivity in the study area is more sensitive to the change in water content. When the water content is lower than the loess liquid limit, the curve shape shows first-order exponential attenuation. When the water content of the soil is very low, a small increase in water content rapidly reduces the resistivity of the sample. This finding is due to the large porosity and the existence of pores in the loess, thereby hindering the conduction of current; as a result, the resistivity increases. However, when the water content in the sample increases, the larger pore in the loess is filled by water rapidly. As the mineralization of groundwater in the study area is high and the conductivity increases greatly, the resistivity of the sample decreases sharply. With the continuous increase in water content, the area of water molecules in soil pore increases, the adsorbed current increases, and the resistivity decreases, but the attenuation rate decreases gradually. When the water content of the soil mass reaches 30% (the liquid limit of the loess in the study area), the increase in water content no longer leads to a remarkably decrease in resistivity. At this time, the saturation in the loess is high, and the pore between the loess particles is filled with water, thereby indicating that the increase in the water content of the soil mass has not improved the conductivity of the samples. Mineralization in groundwater is the main controlling factor of apparent resistivity. According to the relationship between water content and resistivity of loess in the study area, when the liquid limit of loess is reached, the resistivity value is stable at 28.35 Ω m. Therefore, the apparent resistivity corresponding to the groundwater level in the loess layer in the study area is approximately 28.35 Ω m.

### The results of ERT

**Figure 9:**
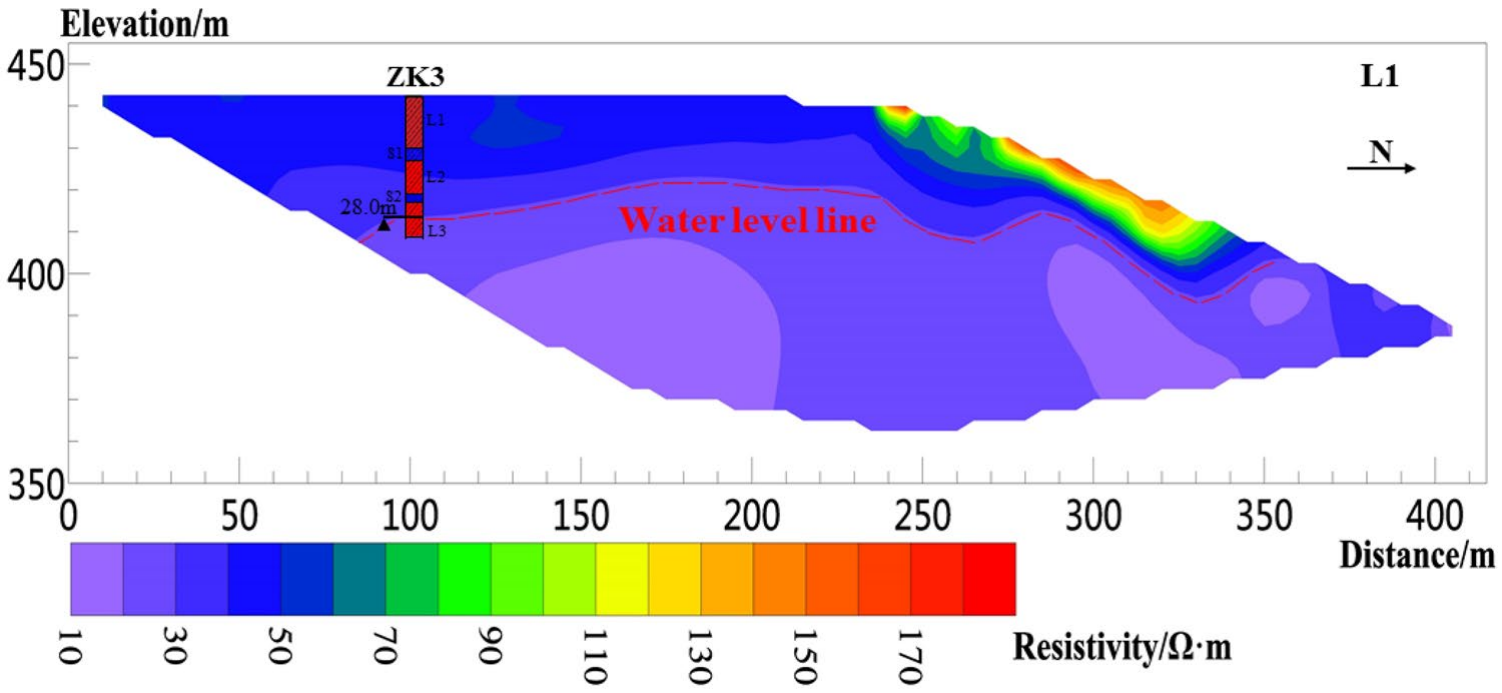
Resistivity profile of line L1.

**Figure 10:**
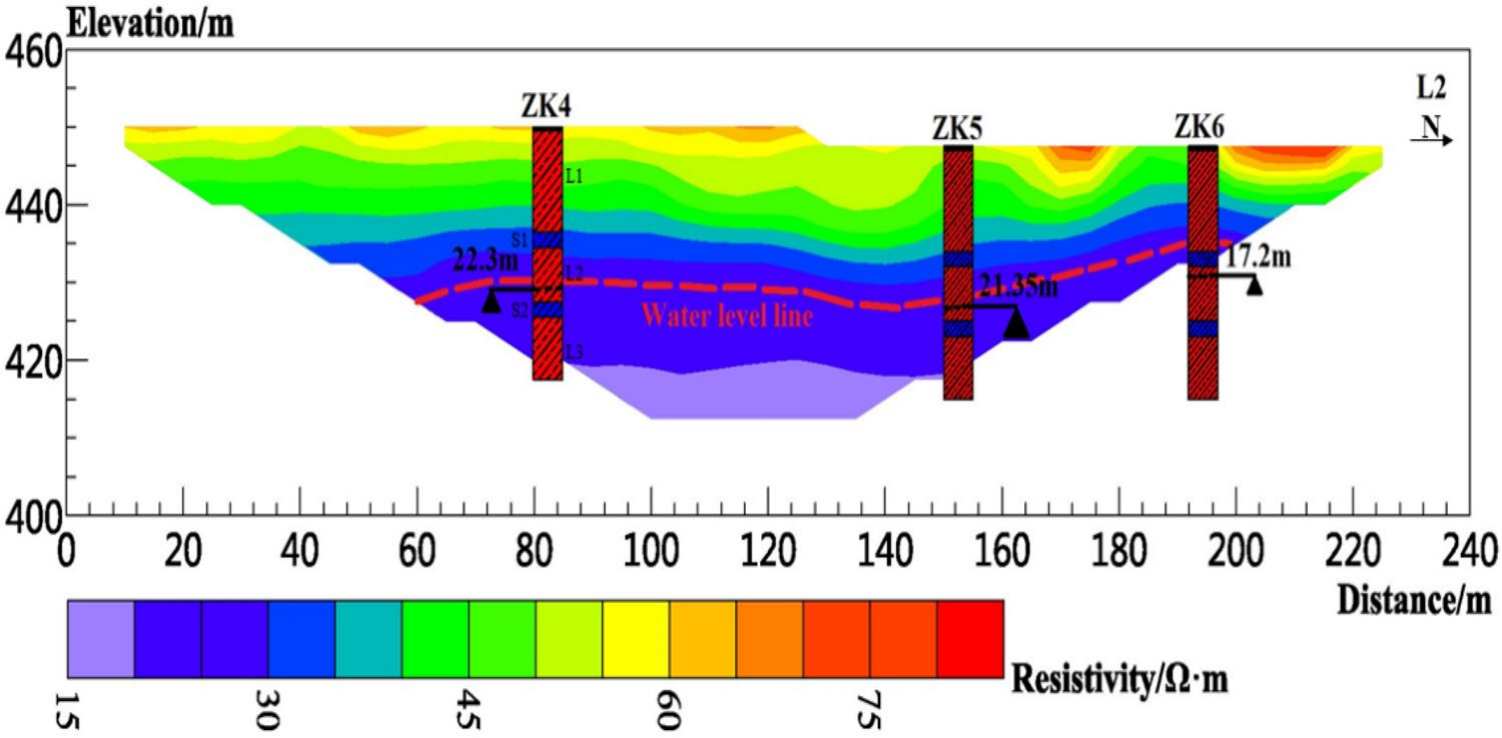
Resistivity profile of line L2.

**Figure 11:**
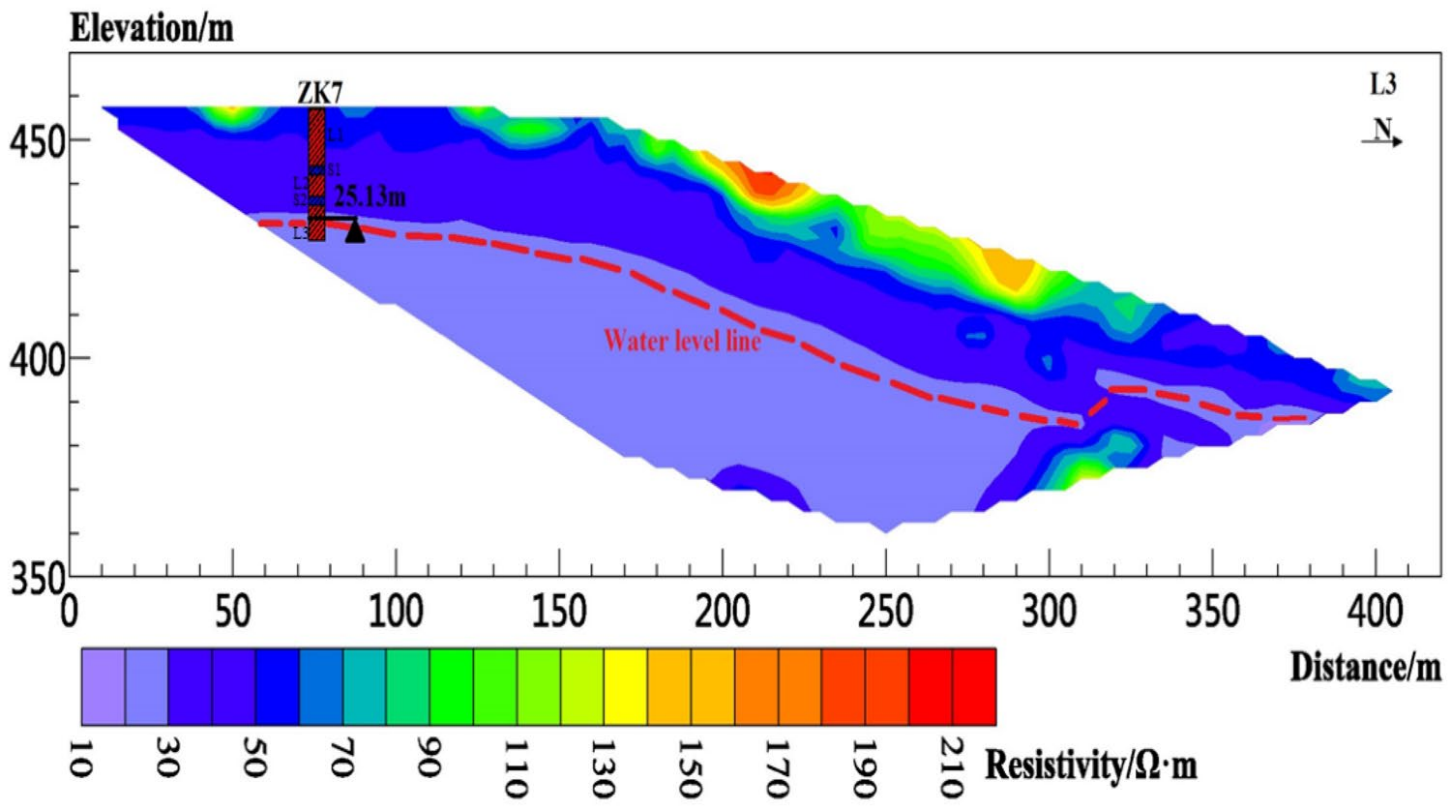
Resistivity profile of line L3.

**Figure 12:**
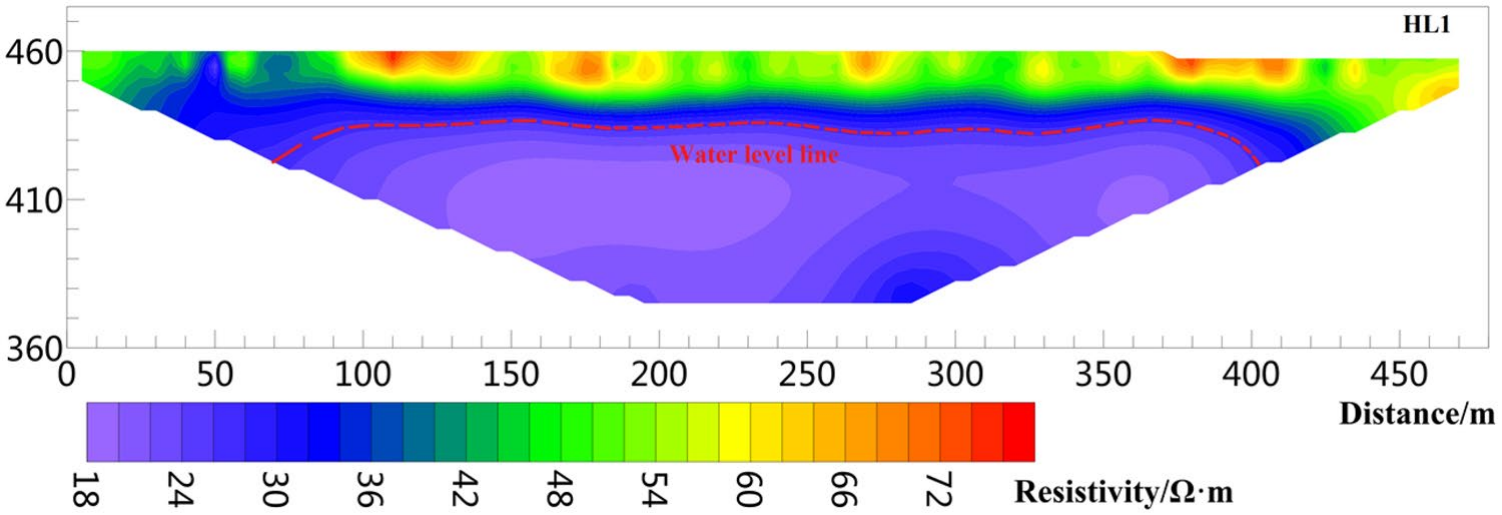
Resistivity profile of line HL1.

**Figure 13:**
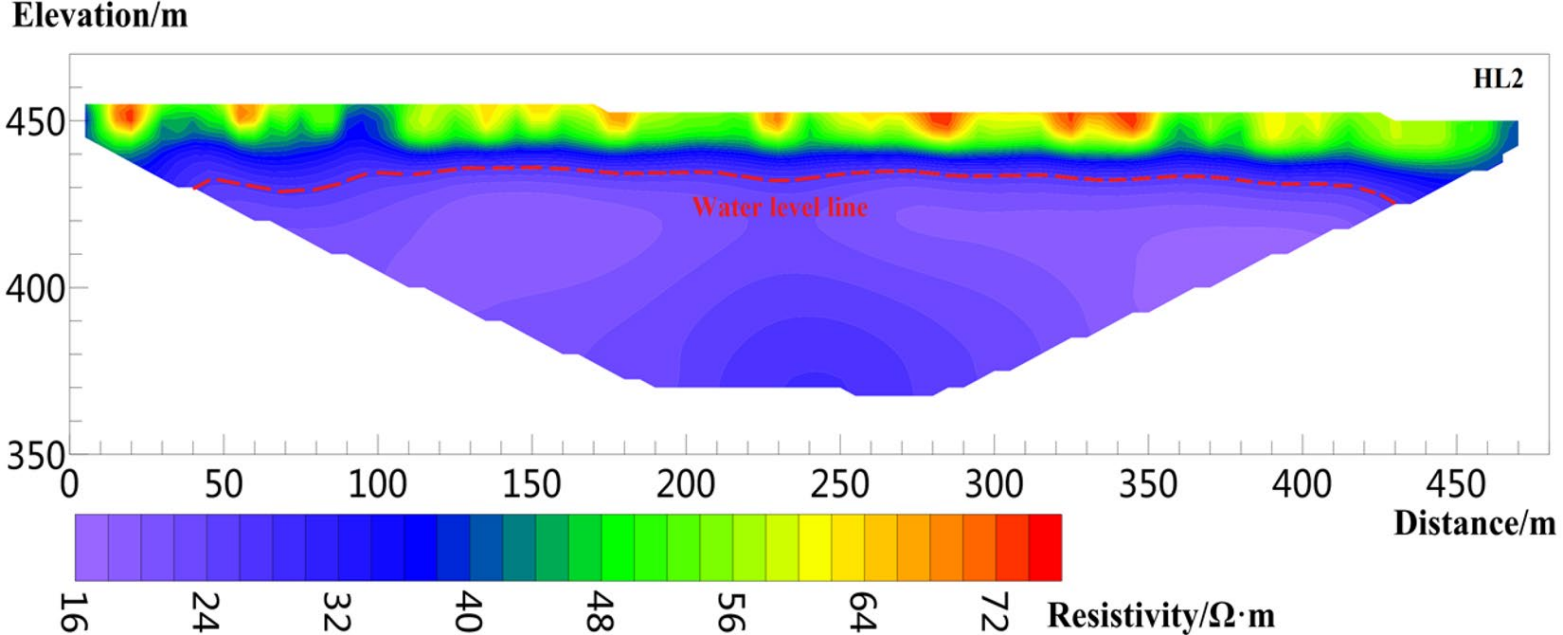
Resistivity profile of line HL2.

**Figure 14:**
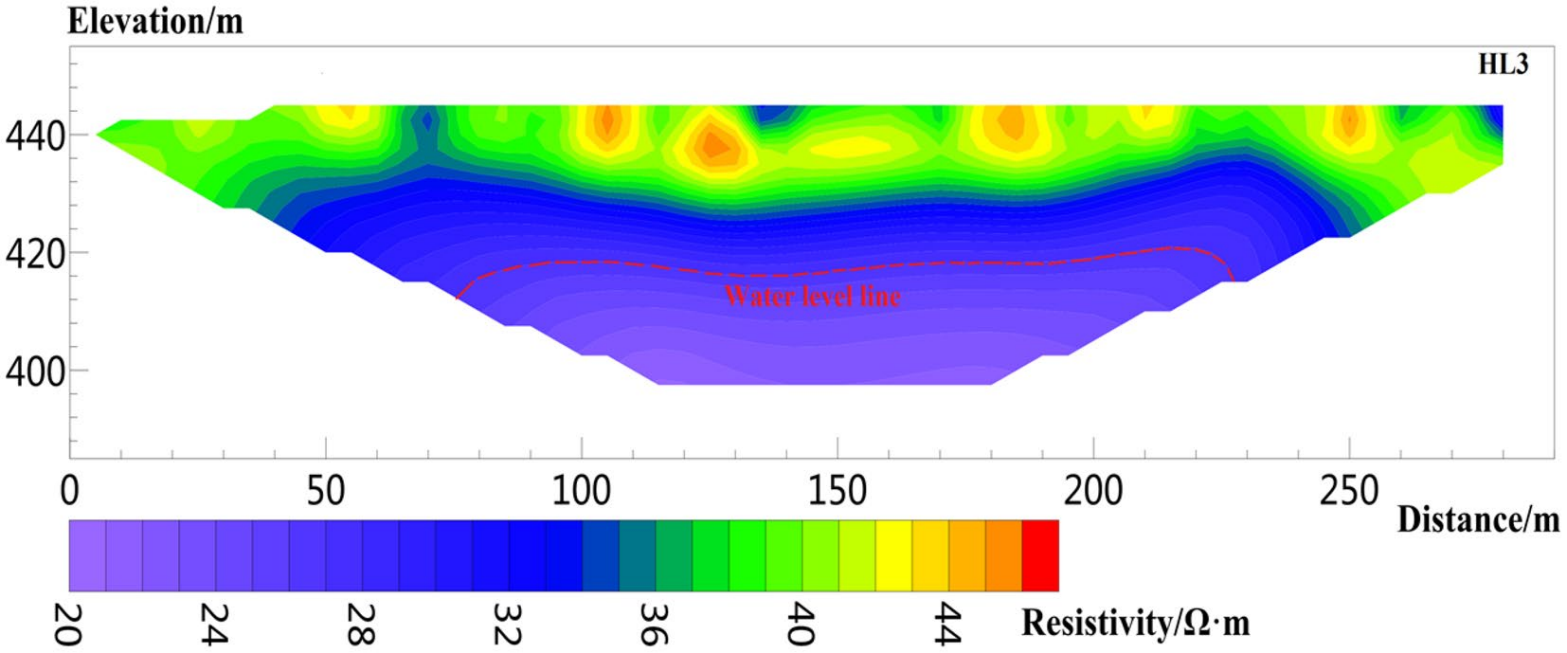
Resistivity profile of line HL3.

Six sections are arranged in the study area (Fig. 1b), of which three sections are perpendicular to the sliding direction of the landslide, i.e., survey lines L1, L2, and L3, as shown in Figs. 8, 9, 10, 11, 12 and 13, and three sections are parallel to the tableland, i.e., transverse survey lines HL1, HL2, and Hl3, as shown in Figs. 9, 10, 11, 12, 13 and 14.

Figure 9 shows the inversion results of line L1, which has been iterated for four times. The fitting error RMS is 3.4%, the line length is 420 m, and the effective detection depth is approximately 90 m. The resistivity range in the figure is approximately 10–200 Ω m. The measuring line length of 0–240 m is arranged above the tableland, 240–340 m is arranged on the slope, and 340–420 m is arranged on the secondary terrace of the Jinghe River under the slope. The resistivity of the 0–20 m shallow layer on the tableland is approximately 40–65 Ω m, and that of the 0–10 m shallow layer on the slope is approximately 40–200 Ω m. Loose macroporous soil particles and gravel deposited after the landslide due to the existence of shrubs in the landslide direction. The resistivity of the 20–28 m formation depth is approximately 28–40 Ω m. According to the laboratory test results, the resistivity of 28.35 Ω m corresponds to the water content of saturated loess. Therefore, 20–28 m is the unsaturated layer above the water table, and the resistivity below the 28 m formation depth is lower than 28 Ω· m, which is inferred as the aquifer.

Figure 10 shows the inversion result of line L2, which has been iterated for five times, the fitting error RMS is 2.8%, the length of the line is 230 m, and the effective detection depth is approximately 40 m. The resistivity range in the figure is approximately 16–120 Ω m, and the resistivity of the shallow layer 0–10 m is high at approximately 60–120 Ω m. When L2 is deployed, a very steep slope is encountered by the survey line L2. The slope is approximately 75°, and the vertical height is approximately 25 m. Therefore, the survey line L2 is only deployed on the tableland, so the resistivity tomography cannot detect the groundwater level on the slope. The resistivity of the formation with a depth of 10–20 m is approximately 28–60 Ω m. This layer is unsaturated above the water table. The resistivity below the formation depth of 20 m is lower than 28 Ω m, which is inferred as an aquifer.

Figure 11 shows the inversion result of L3, which has been iterated four times. The fitting error RMS is 5.6%, the line length is 420 m, and the effective detection depth is approximately 90 m. The apparent resistivity range in the figure is approximately 16–240 Ω m. The measuring line length of 0–160 m is arranged above the tableland, and the resistivity of the 0–10 m shallow layer on the plateau is approximately 45–65 Ω m. The measuring line length of 160–420 m is arranged on the slope, and the apparent resistivity of the 0–15 m shallow layer on the slope is approximately 40–200 Ω m. The reason for the high resistivity is that shrubs growing on the slope and existing gravel hinder the conduction of current. The slope protection works are carried out near L3, and the mixture of compacted soil and concrete is stacked between 200 and 220 m of L3, thereby showing a high resistivity between 200 and 220 m. However, between 270 and 280 m of L3, a roadway provides easy construction for slope protection works. Pedestrians and vehicles compact the soil, resulting in relatively high resistivity. The resistivity of the formation depth of 10–25 m is approximately 28–45 Ω m; thus, the formation is an unsaturated layer above the phreatic aquifer. Resistivity below 25 m is lower than 280 Ω m; it is inferred as an aquifer below 25 m.

Figure 12 shows the result of HL1, which was laid out parallel to the edge of the slope. The number of data iterations is five times, and the RMS is 2%. The length of the line is 480 m, and the effective detection depth is 100 m. The resistivity range is 16–90 Ω m. The shallow layer 0–10 m shows high resistivity, and the resistivity range is approximately 40–90 Ω m. The formation depth is 10–25 m, and the resistivity range is 28–40 Ω m; this layer is inferred unsaturated above the phreatic aquifer. The resistivity below 25 m is less than 28 Ω m, which can be deduced as an aquifer.

Figure 13 shows the result of HL2, where the number of data iterations is five times, and the RMS is 1.8%. The length of the line is 480 m, and the effective detection depth is 100 m. The resistivity range is 14–90 Ω· m. The shallow layer 0–10 m shows high resistivity, and the resistivity range is approximately 40–90 Ω· m. The formation depth is 10–20 m, and the resistivity range is 28–40Ω· m; this layer is inferred unsaturated above the phreatic aquifer. The resistivity below 25 m is less than 28 Ω· m, which can be deduced as an aquifer.

Figure 14 shows the result of HL3, where the number of data iterations is five times, and the RMS is 1.6%. The length of the line is 300 m, and the effective detection depth is 45 m. The resistivity range is 14–70 Ω m. The shallow layer 0–15 m shows high resistivity, and the resistivity range is approximately 40–90 Ω m. The formation depth is 15–28 m, and the resistivity range is 28–40 Ω m; this layer is inferred unsaturated above the phreatic aquifer. The resistivity below 28 m is less than 28 Ω· m, which can be deduced as an aquifer.

The inverted resistivity values in Figs. 9, 10, 11, 12, 13 and 14 show a trend of gradually decreasing from the surface layer to the deep layer and evident stratification characteristics, consistent with the soil stratification characteristics inside the Loess Plateau. Figures 9 and 11 show the surveying lines arranged perpendicular to the direction of the landslide. The law of resistivity variation indicates that the groundwater level begins to drop significantly when approaching the plateau edge until the end of the surveying line. The end of the surveying line is located on the second terrace of the Jinghe River. Therefore, the location from the end of the surveying line to the side of the Jinghe River is the area of groundwater confluence, where the water level is shallow. The downward trend of the groundwater level in Figs. 9 and 11 is consistent with the direction of the landslide, also indicating that the occurrence of loess landslide is closely related to the groundwater level. Figures 12, 13 and 14 show a survey line laid horizontally in the direction of the landslide. The inversion results show that the distribution of the groundwater level in the loess stratum is continuous in the study area, no evident fluctuation trend of groundwater level rising or falling greatly is observed, and the groundwater level changes smoothly. The groundwater level of HL 1 and HL3 is approximately 28.8 and 25.5 m, respectively, and that of HL2 is 21.5 m. The water level of HL2 is shallower than those of HL 1 and HL3. The reason is that the location of HL1 and HL3 is cultivated land with few crops, with little or no irrigation. However, HL2 is located in a peach orchard with a large area of peach trees, large amount of irrigation yearly, and is frequently irrigated. Therefore, the amount of irrigation also affects the buried depth of the groundwater level.

### Verification of borehole data

Boreholes were drilled in the study area to verify and improve the accuracy of electrical resistivity tomography results. The boreholes are located at approximately 100 m (ZK3) from the starting point of L1, 80 (ZK4), 150 (ZK5), and 190 m (ZK6) from the starting point of L2; 80 m (ZK7) from the starting point of L3. Borehole lithology is recorded in Table 1. The borehole location and measured groundwater level depth are shown in Figs. 9, 10 and 11. The orderly intersection of the loess–paleosol sequence in the borehole data in Table 1 is a demonstration of the stratigraphic stratification characteristics within the Loess Plateau, consistent with the typical stratigraphic profile exposed on the Loess Plateau in the study area in Fig. 2. The borehole data show the 0.5 m planting root soil on the surface of the study area. The average thickness of the L1 loess is approximately 13 m, and those of the L2 loess, L3 loess, S1 paleosol, and S2 paleosol are approximately 6, 8, 2.5, and 2 m, respectively.

**Table 1:** Borehole lithology and stratification.

Borehole number	Stratigraphic lithology					
	Planting soil L0 (m)	Loess L1 (m)	Paleosol S1 (m)	Loess L2 (m)	Paleosol S2 (m)	Loess L3 (m)
ZK3	0.5	12	3	8	2	8
ZK4	0.5	13	2	7	2	8
ZK5	0.5	13	2	7	2	8
ZK6	0.5	13	2	7	2	8
ZK7	0.5	13	2	5	2	8

According to the relationship between water content and resistivity of loess obtained from laboratory tests, when the water content of the loess reaches 30% of the liquid limit, the corresponding resistivity is 28.35 Ω m. Therefore, the position of groundwater is estimated in the inversion results. The red dotted line in Figs. 8, 9, 10, 11, 12, 13 and 14 indicates the water level of groundwater inferred by the electrical resistivity tomography, whereas the black dotted line in boreholes 3–7 indicates the actual measured stable water level of groundwater. The comparison results between the measured groundwater level line from the borehole and the estimated groundwater level line from the electrical resistivity tomography are shown in Figs. 8, 9 and 10. Comparison results of the water level line inferred by electrical resistivity tomography and the measured water level line in the borehole are listed in Table 2.

**Table 2:** Comparison between inferred water level of high-density survey line and measured water level of borehole.

Borehole number	Inferred groundwater level (m)	Measured groundwater level (m)	Difference (m)
ZK3	28.8	28	0.8
ZK4	21.6	21.3	0.3
ZK5	21.2	21.35	0.15
ZK6	15.2	17.2	2.2
ZK7	25.5	25.13	0.37

Figure 1b shows that due to site conditions, the location of ZK3 does not completely coincide with L1, and the horizontal distance from L1 is approximately 70 m, thereby resulting in a difference of 0.8 m between the estimated and measured groundwater level depths at ZK3. ZK4–ZK6 exactly coincide with L2. The measured groundwater level depths of ZK4 and ZK5 are almost identical to those of the groundwater level estimated by the electrical resistivity tomography. The reason for the above situation is that the “inverted trapezoid” edge data are interpolated and estimated during data inversion, resulting in the deviation of the inverted data. Moreover, ZK6 is at the rightmost edge of the L2 resistivity profile, leading to a large deviation between the inferred water level and the measured water level. ZK7 does not completely coincide with L3, and the horizontal distance from L3 is approximately 30 m. The estimated depth of the groundwater level is normal compared with the measured water level in the borehole.

In summary, the measured depth of the groundwater level in the borehole does not fully coincide with the depth of the groundwater level estimated by the electrical resistivity tomography. However, through comparative analysis, except for ZK6, the difference between the measured groundwater level depth in other boreholes and the inferred groundwater level depth by the electrical resistivity tomography is within the error requirements. Therefore, the non-intrusive resistivity tomography technology has been proven to have high accuracy in detecting the depth of the groundwater level in the Loess Plateau.

## 3D geological modeling

**Figure 15:**
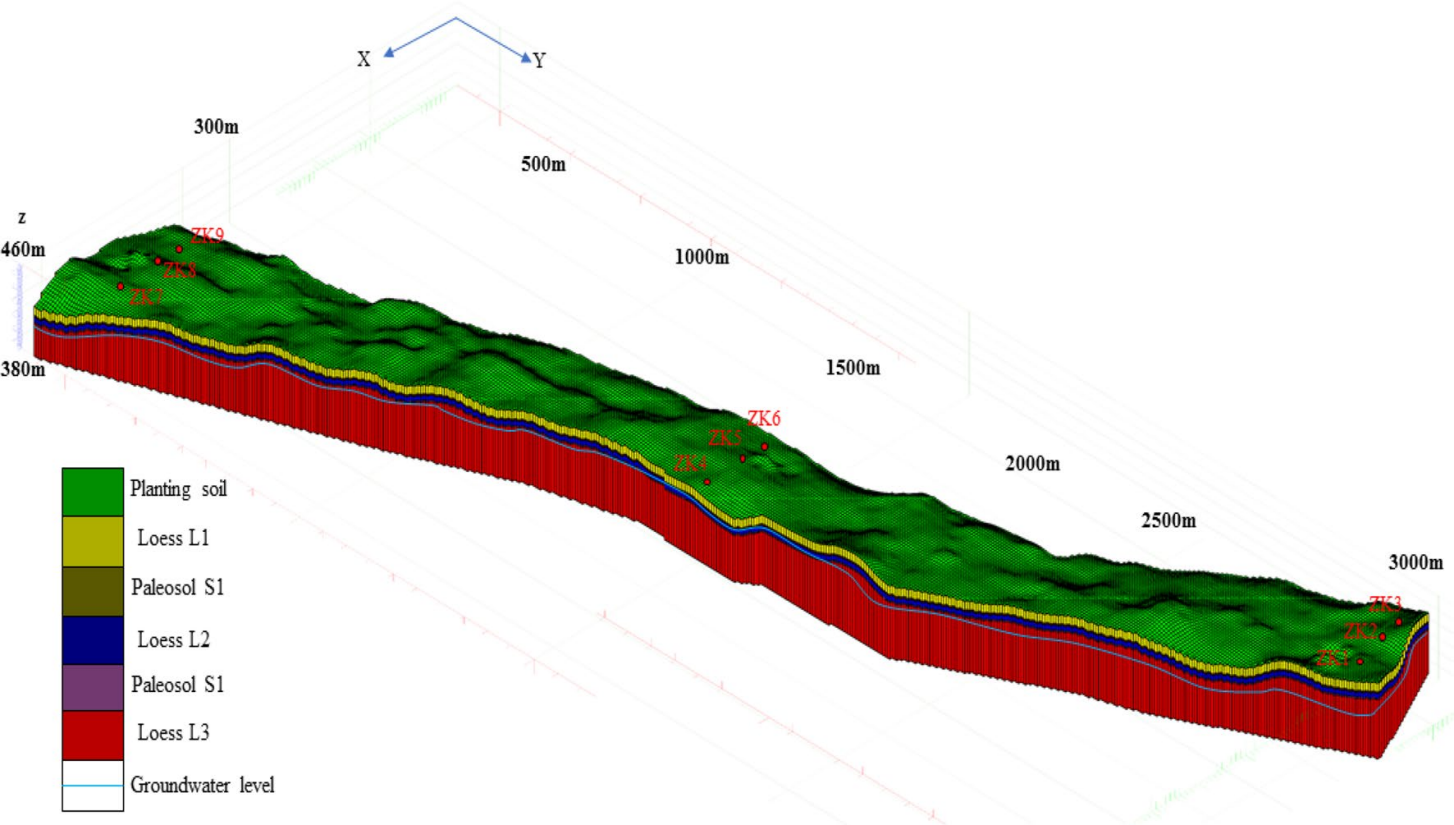
3D geological model.

The 3D geological model is an advantageous method to show the structural and spatial distribution characteristics of landslide in detail^35^; it provides a basis for studying the triggering mechanism of landslide, landslide stability evaluation, and spatio-temporal variation of groundwater level. A joint investigation was carried out to accurately construct the 3D geological model of the study area. The depth of the groundwater table, lithology of different strata, and soil thickness in the Loess Plateau of the study area were determined by combining the borehole and inversion data of the electrical resistivity tomography. At the same time, the change in topographic relief in the study area was determined based on the digital elevation model (DEM) data of 30 m in the study area. The detailed data obtained are used to construct a 3D geological model of the research area, as shown in Fig. 15. The established 3D geological model not only provides technical guidance and basis for the study of groundwater level fluctuation, but also a basis for the stability evaluation of loess slope and the prediction and warning of landslide.

## Conclusion

To addresses the limitation and inaccuracy in determining the stratum structure and groundwater level by solely relying on borehole information or ERT. A combined survey was conducted in the South Jingyang tableland, including the electrical resistivity tomography, borehole, and laboratory tests. Through the analysis of the results, the following conclusions are obtained.

1. The buried depth of the groundwater level in the study area and the internal stratification characteristics of the loess tableland are preliminarily inferred from the inversion results of the survey of the South Jingyang tableland with the electrical resistivity tomography. The results of the vertical survey lines (L1, L2, and L3) indicate that the groundwater level decreases along the landslide direction, consistent with the sliding direction when the landslide occurs. The results also imply that the occurrence of landslide is closely correlated to the groundwater level. Moreover, the amount of irrigation affects the buried depth of the groundwater level, and a large amount of irrigation leads to the rise of the groundwater level.
2. The quantitative relationship between resistivity and water content obtained from laboratory tests clearly demonstrates that the resistivity of the loess in the study area is very sensitive to the change in water content. When the soil water content is very low, a slight increase in water content rapidly reduces the loess resistivity. When the water content of the loess exceeds 30% of the liquid limit, the resistivity hardly changes with the increase in water content. When the water content of the loess reaches 30% of the liquid limit, the corresponding resistivity is 28.35 Ω m, which corresponds to the resistivity of the groundwater level in the loess slope of the South Jingyang tableland.
3. The combined inversion results of the electrical resistivity tomography and those of the laboratory test indicate that the depth of the groundwater level in the plateau is more accurate. At the same time, the borehole lithology data and the measured buried depth of the groundwater level not only verify the effectiveness of the electrical resistivity tomography, but also reveal the internal structure, the thickness of each soil layer, and the distribution and buried depth of the groundwater level in the South Jingyang tableland.
4. On the basis of the results of the electrical resistivity tomography, laboratory test results, and borehole lithology data, a detailed 3D geological model of the study area is constructed. The model more accurately describes the internal structure characteristics of the loess slope and the temporal and spatial distribution characteristics of the groundwater level in the southern tableland of Jingyang. The 3D geological model not only provides a basis for studying the temporal and spatial variation law of the groundwater level, but also a reference for landslide stability analysis, prediction, and early warning.
5. Because the traditional borehole exploration method is time-consuming, laborious, and destroys the internal geological structure of the landslide, combining the geophysical exploration method is recommended when investigating the landslide. This method is not only low cost and highly efficient, but also rich in information.
6. It is suggested that the ERT to accurately locate the buried depth of the groundwater table should be combined with qualitative and quantitative analysis.

## Data Availability

The data used to support the results of the study are included within the paper.
